# The PLN Foundation is striving for a cure, but who owns the disease?

**DOI:** 10.1007/s12471-025-01961-4

**Published:** 2025-05-08

**Authors:** Luuk Kerckhaert, Pieter Glijnis, Annet N. Linders, Cor Oosterwijk, Pieter A. Doevendans

**Affiliations:** 1 PLN Foundation, Wieringerwerf, The Netherlands; 2 VSOP—Patiëntenkoepel voor zeldzame en genetische aandoeningen, Utrecht, The Netherlands; 3 Netherlands Heart Institute, Utrecht, The Netherlands; 4 Department of Cardiology, Division of Heart and Lungs, University Medical Centre Utrecht, Utrecht, The Netherlands

**Keywords:** Intellectual property, Inherited cardiomyopathy, Ownership, Material transfer agreement, Foundation

## Abstract

The PLN Foundation, established in 2012, supports about 1700 individuals with a phospholamban (PLN) gene mutation causing severe cardiomyopathy. It aims to cure this rare disease by collaborating with universities, research institutions, and biotechnology companies. However, the foundation often faces challenges in being recognised as an equal research partner, with legal departments and technology transfer offices (TTOs) prioritising institutional interests over the public good, leading to delays and inefficiencies. The scientific culture’s ‘publish or perish’ mentality, patent ownership issues, and bureaucratic ethics review processes further complicate progress. To overcome these barriers, the foundation advocates IP co-ownership, patient leadership, streamlined agreements, provisional ethical approvals, improved research logistics, revised evaluation metrics for scientists, and a shift in TTO strategies towards co-creation. These measures aim to enhance collaboration, accelerate therapeutic development, and ensure the accessibility and affordability of new treatments for rare diseases.

## Introduction

The PLN Foundation, established in 2012, is a patient-run organisation that represents and supports about 1700 individuals who carry a genetic mutation in the phospholamban (PLN) gene. This mutation, known as PLN c.40_42del or PLN R14del, is associated with a severe form of dilated and/or arrhythmogenic cardiomyopathy, although the phenotype varies significantly among carriers. As a patient organisation, the PLN Foundation is dedicated to developing a cure for this currently incurable orphan disease. In its quest, the foundation successfully collaborates with a broad network of universities, research institutions, and biotechnology companies.

However, patient organisations that operate like the PLN Foundation often face challenges, as they are perceived primarily to be sources of funding, (genetic) information, and materials, rather than equal research partners. Legal departments frequently hinder their efforts to secure ownership of their contributions, even though holding intellectual property (IP) would help these organisations ensure affordable and accessible treatments.

Patient organisations, especially those focused on orphan diseases, can be a powerful driving force in drug development due to their intrinsic motivation to accelerate progress while keeping an eye on accessibility and affordability [1]. Their critical role as a hub in the drug development pathway should be acknowledged through genuine partnerships rather than mere contributions. By facilitating patient recruitment, advising on relevant outcome measures, and collaborating in trial design, these organisations can help shorten trial timelines and potentially expedite regulatory submissions for new therapies.

This article aims to identify the existing challenges within the ecosystem in which patient organisations operate, so that these issues can be addressed to pave the way for equitable collaboration and joint ownership in research partnerships.

## Scientific culture

The current culture within the scientific world presents several obstacles for patient organisations striving to accelerate therapeutic development. While researchers have made remarkable progress in advancing knowledge and understanding of diseases, the structure of the scientific system prioritises publications over developing therapies. Scientists, despite their expertise in fundamental research and discovery, frequently lack awareness of the necessary steps required to advance a medicine through the process of registration and market authorisation.

Operating within a highly competitive field, data sharing typically occurs only after publication. This practice hinders swift drug development and excludes other stakeholders, such as patient organisations.

## Publish or perish cycle

The reality of ‘publish or perish’ remains a dominant force in shaping scientists’ careers. Securing publications in high-ranking journals directly influences scientists’ impact factors or Hirsch indices (H-index) and thereby shapes future funding opportunities [2]. As more funding leads to high-impact publications [3], a significant portion of a scientist’s time is spent obtaining grants to support future work.

This cycle of acquiring funding and securing high-impact publications makes it difficult for scientists to shift focus and dedicate time to creating value from scientific knowledge, especially given the uncertainty of translating discoveries into clinical applications. More support (both practical and financial) from academia and patient organisations can lower the threshold towards valorisation.

## Patent ownership and scientist entrepreneurship

Patents within the European Union (EU) are typically claimed and owned by academic institutions based on scientists’ employment status, often leaving researchers unaware of the possibilities to share ownership or license patents to patient organisations. This obstructs the collaborative efforts needed to advance therapeutic development.

When licensing IP from academic institutions, it is crucial that the institutions follow the principles of socially responsible licensing as published by the Netherlands Federation of University Medical Centres [4], especially when commercial partners are involved. These principles aim to guide knowledge transfer organisations in finding balanced licensing agreements with third parties, ensuring that the IP contributes to society in the most effective way.

## Medical ethics review committees

In line with the challenges presented by the current scientific culture, medical ethics review committees (METCs) compound these difficulties with their bureaucratic processes. While their oversight is crucial for ensuring ethical standards, their fixed meeting schedules and the general lack of flexibility create excessively lengthy review processes.

For example, the SCIENCE trial encountered delays after METC Utrecht demanded additional checks for an already internationally approved protocol, illustrating how these requirements, though well-intentioned, can postpone patient inclusion and negatively affect timely drug development [5]. These procedures significantly slow down research progress and suggest that there is a need for a more flexible and streamlined process to promote drug development.

## Collaboration with academia

Patient organisations, especially ones like the PLN Foundation, often face challenges in gaining recognition as scientific collaborators in the research ecosystem surrounding their specific disease. Despite their expertise and ability to help set up successful scientific collaborations and clinical studies, they are frequently relegated to a fundraising role without receiving full acknowledgement for their vital contributions. Two academic entities are particularly prominent in this issue.

### Legal departments

Legal departments of universities and research institutions play a key role in setting up equitable collaborations but often slow progress with complex processes for material transfer agreements (MTAs) and joint ownership contracts. As publicly funded entities, they could better serve public interests by streamlining these procedures. Numerous cases experienced by the PLN Foundation highlight the need for more efficient, patient-focused legal frameworks to improve research outcomes.

For example, the PLN Foundation had to invest in a mobile laboratory for blood collection, processing, and storage to freely share patient samples with researchers worldwide, to avoid the lengthy discussions surrounding MTAs. This costly investment was necessary even though universities already possess the required facilities and infrastructure.

In another case, the PLN Foundation, despite providing funding and intellectual support, has been locked in negotiations for 4 years with six legal professionals representing a university to gain joint ownership of the project’s results. This drawn-out process has forced the foundation to redirect a significant portion of its funds towards legal advice and support, at the cost of investments in much-needed research efforts.

These experiences highlight how the current approach of academic legal departments, prioritising institutional ownership over the public good, can hinder and delay the advancement of potentially life-saving research.

### Technology transfer offices

Similar to the legal departments, university technology transfer offices (TTOs) largely act in the financial interest of their employer instead of serving the interests of society, as highlighted in a recent debate in the Netherlands [6]. When it comes to therapeutic development for orphan diseases, the profit model is limited or even negative, yet TTOs often demand significant compensation early on for sharing breakthroughs and IP. Their approach prioritises maximising short-term returns without enabling additional investments and exploitation of the IP, which was initially developed with crucial public or patient-organisation funding.

The PLN Foundation argues that since patient organisations invest time, money, and intellectual input into research, they should own a portion of the resulting IP and should be able to develop it in synergy with academia.

Collaboration between academia and patient organisations can be highly productive when structured as true partnerships, with contributions from both sides being fully recognised. In a joint project with Stanford University, where a PLN mutation-specific siRNA was developed, researchers acknowledged the PLN Foundation’s role in funding and intellectual input, resulting in a 50% share of the patent [7]. Similarly, in a collaboration with the Technical University of Munich on a PLN R14del pig model, the foundation’s contributions were recognised through receipt of a significant share of the patent. These examples show how recognising patient organisations’ roles can foster equitable collaborations benefiting research and patient communities.

European universities should shift from technology transfer to co-creation with industry and patient organisations [8]. By adopting flexible IP policies, these institutions can encourage entrepreneurship and improve the societal impact of innovations, particularly in the biotechnology sector where valorisation rates are low [9]. A focus on interdisciplinary collaboration and clear commercialisation strategies can help universities align economic success with broader societal benefits.

A case experienced by the PLN Foundation highlights the challenges of the current valorisation system. After funding promising research, the university’s TTO demanded high licensing fees and filed a patent without consulting the foundation, despite its intellectual input [10]. A patent attorney later confirmed the foundation’s right to be included. This case emphasises the need for fair collaboration between patient organisations and universities, ensuring that those who contribute significantly to research receive a fair stake in the resulting IP.

## Strategy of the PLN Foundation

The PLN Foundation has effectively tackled funding challenges for research on the orphan disease PLN cardiomyopathy by initially funding projects with direct contributions from patients and their communities. This initial funding enables the launch of new projects and the generation of pilot data, which are crucial for applying for larger, more competitive grants. To streamline the grant-writing process, the PLN Foundation collaborates with specialised companies and scientists, improving their success in acquiring funds for both basic and pre-clinical research [11–13]. These efforts have laid the groundwork for critical advancements in the understanding and treatment of PLN cardiomyopathy.

A central goal of the PLN Foundation is to develop an affordable cure for individuals affected by the PLN R14Del mutation. Given the typically high costs associated with therapies for orphan diseases, the foundation prioritises acquiring at least partial ownership of the IP. In addition to funding and providing materials, the PLN Foundation secures IP by offering crucial input to the projects it supports, leveraging its extensive research network and advisers who are experts in the field.

While organisations that use a venture philanthropy model, such as CureDuchenne and the Cystic Fibrosis Foundation [14], often leverage IP co-ownership primarily for royalty income, the PLN Foundation aims to use its stake to negotiate prices that fit Dutch healthcare reimbursement standards. This strategy bolsters its position when partnering with pharmaceutical companies, which are essential partners in developing and distributing new therapies [15], and also ensures that affordability and accessibility remain central.

Despite the challenges, the PLN Foundation remains deeply grateful for its collaborations with excellent researchers worldwide, which have led to significant breakthroughs and novel therapeutic strategies. These partnerships have raised global awareness of PLN R14Del cardiomyopathy and attracted numerous biotechnology and pharmaceutical companies working toward a cure.

The purpose of this article is therefore not to criticise individual researchers, but rather to suggest improvements to the research ecosystem in which they operate, ensuring that their groundbreaking work can achieve the full impact it deserves.

## Conclusion and recommendations

In conclusion, the PLN Foundation’s experience underscores the critical need for more equitable partnerships between patient organisations and academic institutions in the quest for therapeutic advancements. Recognising and addressing the systemic barriers, such as bureaucratic delays and rigid IP ownership policies, can enhance collaboration and accelerate the development of life-saving treatments.

To foster these improvements, we propose the following recommendations based on the insights gathered:

- *IP co-ownership:* Encourage (co-)ownership of IP by patient organisations to ensure that their interests are represented in the development and commercialisation of therapies.
- *Patient leadership in orphan disease research*: Enable patients to steer initiatives by setting priorities, coordinating collaborations, and defining outcomes—without necessarily performing bench work. For instance, the European Patients’ Academy trains patients to actively engage in research, ensuring their perspectives and needs drive innovation while managing expectations realistically.
- *Standardised EU MTA*: Develop and implement a standardised EU MTA to streamline the sharing of materials and data, thereby reducing bureaucratic delays and enabling more dynamic collaborative research.
- *Provisional METC approval*: Allow METCs to grant provisional approvals when only minor issues are pending, to accelerate research progress without compromising ethical standards.
- *Better research logistics*: Optimise research logistics by centralising research infrastructure and streamlining administrative processes. These steps can reduce duplication, lower costs, and make research more efficient.
- *Revised evaluation metrics for scientists*: Modify the criteria for evaluating scientific success to include the impact of research on disease treatment and job creation, in addition to traditional publication metrics. This would incentivise scientists to focus on translational and clinical outcomes that have a direct societal benefit.
- *Collaborative efforts with legal departments*: Foster a more cooperative approach between patient organisations and university legal departments to ensure that contract and IP negotiations are conducted with an emphasis on fairness and mutual benefit, rather than solely institutional gain.
- *TTO strategy revision*: Encourage TTOs to shift from a profit-maximising approach to one that emphasises co-creation and long-term value generation, particularly in the context of treatments for orphan diseases.

These recommendations aim to cultivate a research environment that is not only more inclusive and patient-oriented but also more effective in translating scientific discoveries into accessible and affordable health solutions.
